# Supplementary material for: Small Molecule Docking of DNA Repair Proteins Associated with Cancer Survival Following PCNA Metagene Adjustment: A Potential Novel Class of Repair Inhibitors
Source: Molecules. 2019 Feb 12;24(3):645. doi: 10.3390/molecules24030645 (PMC6384788; doi:10.3390/molecules24030645)
Supplement: Supplementary file 1 [file molecules-24-00645-s001.pdf]

Table S1. GBM: List of physio-chemical properties and predicted toxicology and ADME for top 20 ligands. Toxicology and ADME predictions are probabilities in the range [0,1]*.

Name	MW	LogP	LogS	TPSA	Rotb	HBD	HBA	BBB	HIA	HERG	AMES	FHM	HBT	TPT	CYP1A2	CYP2C9	CYP2C19	CYP2D6	CYP3A4
626388	384.47	4.08	-5.84	37.38	1	1	2	0.94	0.99	0.1	0.1	0	0.02	0.98	0.37	0.52	0.7	0.37	0.64
652525	382.8	3.55	-5.29	63.6	1	1	4	0.94	1	0.47	0.47	0.89	0.98	0.95	0.56	0.71	0.71	0.28	0.53
634724	392.36	3.01	-4.39	82.06	1	1	6	0.63	0.99	0.5	0.5	1	0.99	0.95	0.53	0.71	0.76	0.31	0.72
641172	388.37	4.41	-5.3	100.9	4	2	5	0.92	0.93	0.29	0.29	1	0.7	0.95	0.41	0.19	0.27	0.13	0.19
9858	329.44	4.44	-5.36	75.89	2	3	1	1	0.97	0.11	0.11	1	0.77	0.96	0.5	0.17	0.2	0.35	0.25
660032	331.32	2.21	-5.1	35.53	0	1	4	0.05	0.97	0.34	0.34	0.08	0.98	0.95	0.87	0.72	0.89	0.38	0.79
39310	244.31	3.26	-5.9	0	0	0	0	1	0.98	0.08	0.08	0.31	0.36	0.95	0.82	0.1	0.41	0.28	0.21
606174	392.36	3.01	-4.39	82.06	1	1	6	0.63	0.99	0.5	0.5	1	0.99	0.95	0.53	0.71	0.76	0.31	0.72
690391	391.38	2.69	-4.7	84.86	1	2	6	0.03	0.95	0.29	0.29	0.13	0.94	0.95	0.31	0.64	0.83	0.28	0.81
326757	318.32	3.93	-5.21	74.6	4	2	4	0.98	0.98	0.05	0.05	1	0.53	0.98	0.52	0.18	0.29	0.18	0.12
613799	347.37	2.6	-5.1	66.4	1	2	4	0.06	0.97	0.24	0.24	0	0.94	0.95	0.31	0.49	0.65	0.14	0.64
369395	363.37	2.19	-4.38	89.62	1	2	4	0.9	1	0.35	0.35	0.36	0.99	0.95	0.56	0.52	0.55	0.34	0.6
603071	363.37	2.19	-4.38	89.62	1	2	4	0.9	1	0.35	0.35	0.36	0.99	0.95	0.56	0.57	0.56	0.35	0.66
603073	363.37	2.19	-4.38	89.62	1	2	4	0.67	1	0.35	0.35	0.36	0.99	0.95	0.59	0.57	0.52	0.28	0.56
673596	392.4	3.34	-4.85	83.83	2	2	5	0.13	1	0.2	0.2	0.99	0.99	0.95	0.53	0.79	0.55	0.26	0.63
94600	348.35	2.92	-4.79	63.6	1	1	4	0.9	1	0.35	0.35	0.36	0.99	0.95	0.56	0.57	0.55	0.28	0.53
10460	287.36	2.92	-4.13	75.89	2	3	1	1	0.97	0.1	0.1	1	0.83	0.98	0.52	0.17	0.22	0.44	0.17
96641	251.29	0.42	-4.44	61.9	2	3	3	1	0.99	0.11	0.11	1	0.1	0.89	0.91	0.11	0.49	0.43	0.28
652524	363.37	2.19	-4.38	89.62	1	2	4	0.9	1	0.35	0.35	0.36	0.99	0.95	0.56	0.52	0.55	0.34	0.6
688500	366.44	1.31	-4	41.9	2	0	4	0.31	0.99	0	0	0	0.34	0.95	0.7	0.93	0.8	0.22	0.72

*Listed are NSC-626388, NSC-652525, NSC-634724, NSC-641172, NSC-9858, NSC-660032, NSC-39310, NSC-606174, NSC-690391, NSC-326757, NSC-613799, NSC-369395, NSC-603071, NSC-603073, NSC-673596, NSC-94600, NSC-10460, NSC-96641, NSC-652524, and NSC-688500. Receptor binding energies, promiscuity, genotoxicity, skin sensitivity, and aquatic toxicity from SMARTS hits not listed.

Table S2. Melanoma: List of physio-chemical properties and predicted toxicology and ADME for top 20 ligands. Toxicology and ADME predictions are probabilities in the range [0,1]*.

Name	MW	LogP	LogS	TPSA	Rotb	HBD	HBA	BBB	HIA	HERG	AMES	FHM	HBT	TPT	CYP1A2	CYP2C9	CYP2C19	CYP2D6	CYP3A4
713198	389.32	2.16	-5.86	143.46	4	2	5	1	0.7	0.82	0.82	1	0.05	0.98	0.63	0.35	0.31	0.01	0.33
667933	387.39	1.04	-2.97	124.3	5	4	7	1	0.98	0.5	0.5	1	0.53	0.94	0.43	0.54	0.62	0.42	0.35
679749	314.29	4.62	-5.23	68.28	1	0	4	1	0.93	0.02	0.02	1	0.91	0.98	0.52	0.27	0.47	0.08	0.38
707982	390.47	3.63	-5.73	38.77	5	1	3	0.99	0.99	0.49	0.49	0.9	0.56	0.95	0.2	0.36	0.32	0.1	0.39
745321	342.82	2.78	-3.85	56.13	0	1	3	0.25	0.98	0.69	0.69	0	0	0.98	0.37	0.66	0.62	0.33	0.6
714363	351.4	-0.03	-4.8	70.5	3	2	5	0.99	0.97	0.42	0.42	0	0.09	0.96	0.82	0.35	0.64	0.35	0.68
643416	360.41	3.22	-5.28	46.61	2	1	3	0.63	0.99	0.08	0.08	0	0.02	0.98	0.51	0.6	0.75	0.33	0.68
380856	366.37	4.46	-4.93	103.39	2	0	3	0.07	0.96	0.24	0.24	0	0.52	0.96	0.74	0.22	0.84	0.3	0.38
649750	334.33	-0.28	-5.7	41.46	3	1	4	0.95	0.27	0.4	0.4	1	0.59	0.95	0.95	0.36	0.94	0.23	0.24
27034	290.23	3.06	-3.39	122.18	0	2	6	0.58	0.98	0.07	0.07	0.99	0.83	0.95	0.43	0.19	0.35	0.14	0.12
656160	299.71	3.92	-5.59	35.53	1	1	3	0.78	0.97	0.27	0.27	0.04	0.05	0.95	0.89	0.38	0.85	0.38	0.7
690040	384.58	2.74	-3.65	41.13	1	2	3	0.98	0.98	0.78	0.78	0.18	0.43	0.51	0.08	0.11	0.46	0.65	0.28
672425	334.33	1.8	-5.03	80.39	3	2	5	0.5	0.97	0.18	0.18	0	0.2	0.95	0.53	0.56	0.7	0.15	0.41
750908	367.31	1.68	-4.19	146.29	7	3	3	0.42	0.98	0.18	0.18	1	0.76	0.95	0.66	0.45	0.77	0.03	0.43
643735	334.33	1.8	-5.03	80.39	3	2	5	0.5	0.97	0.18	0.18	0	0.2	0.95	0.53	0.56	0.7	0.15	0.41
677959	388.59	2.85	-1.52	46.94	1	2	4	0.93	0.95	0.05	0.05	0.91	0.75	0.45	0.01	0.04	0.06	0.11	0.04
194618	304.3	4.82	-5.23	74.6	0	2	4	0.92	0.98	0.12	0.12	0.92	0.7	0.98	0.7	0.19	0.26	0.2	0.13
656159	283.25	3.7	-5.31	35.53	1	1	3	0.68	0.97	0.61	0.61	0.91	0.18	0.95	0.92	0.23	0.74	0.39	0.69
658142	351.36	0.56	-2.73	81.08	2	2	5	0.31	0.98	0.06	0.06	0.04	0.68	0.98	0.55	0.54	0.8	0.27	0.49
656204	335.38	3.52	-3.43	83.83	0	2	3	0.98	0.33	0.84	0.84	0	0.13	0.33	0.46	0.87	0.89	0.19	0.56

*Listed are NSC-713198, NSC-667933, NSC-679749, NSC-707982, NSC-745321, NSC-714363, NSC-643416, NSC-380856, NSC-649750, NSC-27034, NSC-656160, NSC-690040, NSC-672425, NSC-750908, NSC-643735, NSC-677959, NSC-194618, NSC-656159, NSC-658142, and NSC-656204. Receptor binding energies, promiscuity, genotoxicity, skin sensitivity, and aquatic toxicity from SMARTS hits not listed.

Table S3. Ovarian: List of physio-chemical properties and predicted toxicology and ADME for top 20 ligands. Toxicology and ADME predictions are probabilities in the range [0,1]*.

Name	MW	LogP	LogS	TPSA	Rotb	HBD	HBA	BBB	HIA	HERG	AMES	FHM	HBT	TPT	CYP1A2	CYP2C9	CYP2C19	CYP2D6	CYP3A4
690273	364.35	3.69	-5.74	130.58	7	2	3	0.99	0.88	0.04	0.04	0.76	0.09	0.89	0.86	0.26	0.32	0.05	0.4
720028	303.31	2.82	-5.95	37.3	2	1	3	0.99	0.98	0.05	0.05	0.98	0.37	0.98	0.79	0.48	0.42	0.13	0.29
720447	278.31	2.32	-5.3	34.14	2	2	2	0.99	0.94	0.01	0.01	1	0.99	0.98	0.85	0.53	0.36	0.02	0.25
720702	332.39	4.3	-4.88	35.53	4	0	3	1	0.96	0.01	0.01	1	0.99	0.94	0.49	0.13	0.2	0.1	0.2
753018	378.82	0.05	-5.77	44.62	4	2	5	0.97	0.99	0.3	0.3	0	0.07	0.88	0.88	0.63	0.65	0.11	0.44
722392	270.28	3.47	-3.01	52.6	0	0	4	1	0.96	0.02	0.02	1	0.98	0.94	0.63	0.29	0.44	0.04	0.39
634398	326.41	3.87	-5.47	83.5	4	2	2	0.94	1	0.02	0.02	0	0.76	0.98	0.48	0.47	0.66	0.19	0.36
690678	369.5	2.4	-4.58	55.64	4	1	1	1	0.97	0.06	0.06	0.17	0.43	0.98	0.73	0.7	0.8	0.53	0.58
675223	309.28	0.53	-4.14	97.29	4	1	3	0.92	0.99	0.03	0.03	0	0.08	0.89	0.84	0.46	0.54	0.04	0.39
656204	335.38	3.52	-3.43	83.83	0	2	3	0.98	0.33	0.84	0.84	0	0.13	0.33	0.46	0.87	0.89	0.19	0.56
621457	332.18	2.05	-5.97	46.25	2	2	3	0.37	0.98	0.5	0.5	0.01	0.05	0.89	0.85	0.31	0.72	0.26	0.41
726393	323.34	3.76	-5.89	47.89	5	0	4	0.99	0.94	0.11	0.11	1	0.66	0.94	0.68	0.08	0.34	0.18	0.18
692229	256.25	3.38	-2.82	63.6	1	1	4	0.54	0.98	0.02	0.02	1	0.98	0.94	0.36	0.36	0.59	0.03	0.57
257450	316.26	2.61	-2.49	124.29	1	4	7	0.99	0.96	0.09	0.09	0.98	0.53	0.95	0.54	0.21	0.44	0.1	0.18
116536	282.25	2.83	-4.65	121.35	3	2	2	1	0.98	0.61	0.61	0.76	0.02	0.96	0.76	0.34	0.5	0.06	0.27
70811	278.31	4.49	-5.41	54.18	3	1	4	0.93	0.95	0.05	0.05	0.56	0.82	0.97	0.79	0.11	0.29	0.22	0.14
117192	271.7	3.33	-5.3	53.49	2	2	2	1	0.98	0.77	0.77	0.84	0	0.96	0.76	0.5	0.72	0.12	0.24
753598	365.33	0.18	-5.45	38.33	6	1	3	0.9	1	0.14	0.14	0	0.18	0.32	0.84	0.12	0.41	0.02	0.67
72881	254.28	3.14	-3.44	61.69	4	2	3	0.98	0.95	0.16	0.16	0.45	0.15	0.95	0.7	0.2	0.24	0.11	0.18
5332	249.27	2.93	-5.01	44.95	2	1	4	0.84	0.89	0.1	0.1	1	0.91	0.97	0.78	0.15	0.17	0.08	0.16

*Listed are NSC-690273, NSC-720028, NSC-720447, NSC-720702, NSC-753018, NSC-722392, NSC-634398, NSC-690678, NSC-675223, NSC-656204, NSC-621457, NSC-726393, NSC-692229, NSC-257450, NSC-116536, NSC-70811, NSC-117192, NSC-753598, NSC-72881, and NSC-5332. Receptor binding energies, promiscuity, genotoxicity, skin sensitivity, and aquatic toxicity from SMARTS hits not listed.

Table S4. Renal papillary: List of physio-chemical properties and predicted toxicology and ADME for top 20 ligands. Toxicology and ADME predictions are probabilities in the range [0,1]*.

Name	MW	LogP	LogS	TPSA	Rotb	HBD	HBA	BBB	HIA	HERG	AMES	FHM	HBT	TPT	CYP1A2	CYP2C9	CYP2C19	CYP2D6	CYP3A4
606174	392.36	3.01	-4.39	82.06	1	1	6	0.63	0.99	0.5	0.5	1	0.99	0.95	0.53	0.71	0.76	0.31	0.72
668836	390.52	4.05	-4.62	37.38	1	1	2	0.94	0.99	0.08	0.08	0	0.16	0.98	0.3	0.39	0.53	0.29	0.46
696921	368.35	-1.84	-3.04	133.27	4	1	5	0.19	0.97	0.09	0.09	1	0.17	0.33	0.64	0.2	0.44	0.14	0.12
682298	347.37	2.68	-5.01	69.39	1	1	4	0.71	0.98	0.35	0.35	0	0.99	0.95	0.75	0.72	0.54	0.19	0.53
766292	365.36	-0.99	-5.93	29.1	6	1	4	0.21	0.41	0.56	0.56	0.02	0.02	0.95	0.76	0.5	0.66	0.43	0.54
249910	382.8	3.55	-5.29	63.6	1	1	4	0.94	1	0.47	0.47	0.89	0.98	0.95	0.56	0.7	0.71	0.2	0.64
766871	332.32	-3.09	-4.41	49.33	4	2	6	0.26	1	0.05	0.05	0	0.05	0.88	0.68	0.42	0.48	0.07	0.49
702015	389.4	3.25	-4.87	68.2	4	1	4	0.96	0.8	0.48	0.48	0	0.07	0.96	0.27	0.57	0.6	0.19	0.62
682571	292.33	2.82	-5.64	34.14	2	2	2	0.99	0.94	0.01	0.01	1	0.99	0.98	0.85	0.59	0.45	0.01	0.27
726184	313.26	-0.52	-4.38	69.91	1	0	3	1	0.99	0.17	0.17	1	0.85	0.95	0.91	0.11	0.41	0.15	0.28
683608	311.3	-1.56	-3.01	120.37	6	4	5	0.65	0.99	0.29	0.29	1	0.12	0.95	0.41	0.23	0.22	0.21	0.24
756420	353.39	-1.02	-4.16	46.25	3	2	4	0.85	0.97	0.05	0.05	0	0.04	0.95	0.71	0.2	0.23	0.38	0.17
751644	354.83	1.79	-5.33	41.13	4	2	3	0.99	0.98	0.02	0.02	0	0.05	0.89	0.44	0.62	0.61	0.26	0.52
757177	313.74	-0.06	-5.2	67.15	4	3	3	1	0.95	0.28	0.28	1	0.02	0.93	0.8	0.19	0.58	0.31	0.43
407807	390.51	3.29	-1.5	86.99	1	3	4	0.79	0.91	0.02	0.02	1	0.97	0.69	0.02	0.02	0.07	0.01	0.04
726364	352.38	3.24	-3.76	71.36	4	1	5	0.49	0.67	0.13	0.13	0	0.45	0.95	0.13	0.53	0.35	0.12	0.51
756350	345.36	-2.11	-5.21	33.62	5	1	6	0.99	0.98	0.2	0.2	0.44	0.27	0.74	0.92	0.34	0.43	0.14	0.62
772545	381.43	-2.45	-4.2	44.81	6	2	4	0.83	0.95	0.21	0.21	1	0.07	0	0.8	0.25	0.5	0.16	0.57
626735	308.37	3.1	-4.39	49.74	3	0	3	0.86	0.98	0.17	0.17	1	0.3	0.98	0.17	0.29	0.3	0.75	0.28
729037	311.34	0.36	-2.61	110.73	1	4	3	0.28	0.99	0.14	0.14	0	0.69	1	0.61	0.19	0.3	0.08	0.21

*Listed are NSC-606174, NSC-668836, NSC-696921, NSC-682298, NSC-766292, NSC-249910, NSC-766871, NSC-702015, NSC-682571, NSC-726184, NSC-683608, NSC-756420, NSC-751644, NSC-757177, NSC-407807, NSC-726364, NSC-756350, NSC-772545, NSC-626735, and NSC-729037. Receptor binding energies, promiscuity, genotoxicity, skin sensitivity, and aquatic toxicity from SMARTS hits not listed.

Figure S1. 2D structures of top 20 drug-like ligands for GBM.*

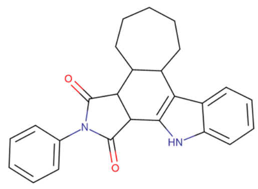

626388

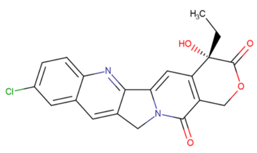

652525

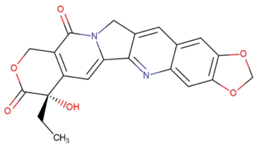

634724

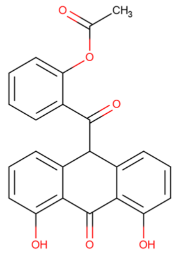

641172

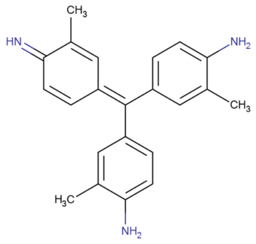

9858

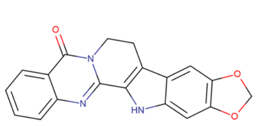

660032

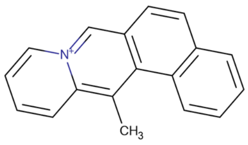

39310

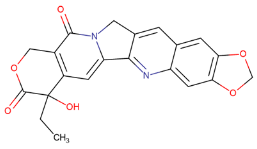

606174

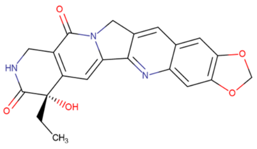

690391

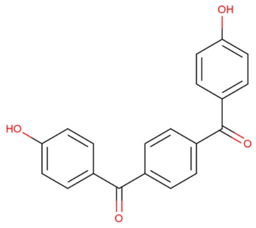

326757

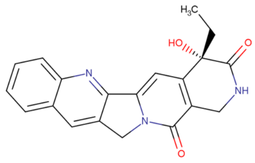

613799

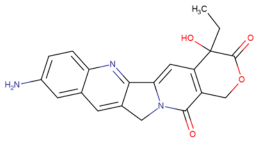

369395

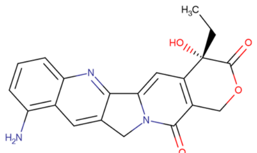

603071

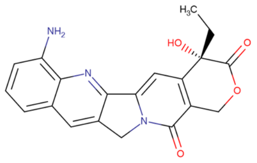

603073

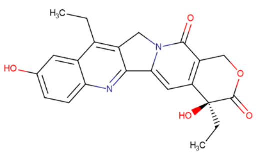

673596

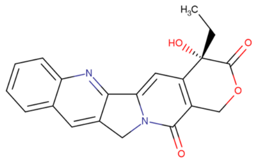

94600

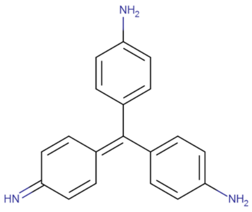

10460

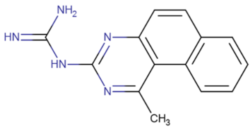

96641

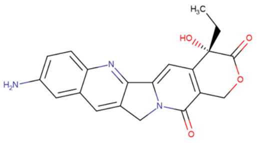

652524

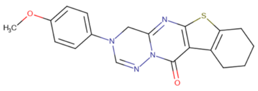

688500

*Listed are NSC-626388, NSC-652525, NSC-634724, NSC-641172, NSC-9858, NSC-660032, NSC-39310, NSC-606174, NSC-690391, NSC-326757, NSC-613799, NSC-369395, NSC-603071, NSC-603073, NSC-673596, NSC-94600, NSC-10460, NSC-96641, NSC-652524, and NSC-688500.

Figure S2. 2D structures of top 20 drug-like ligands for melanoma.*

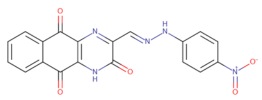

713198

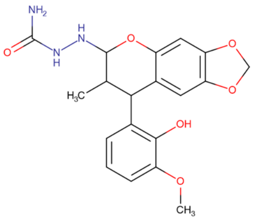

667933

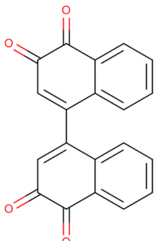

679749

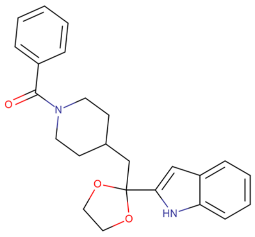

707982

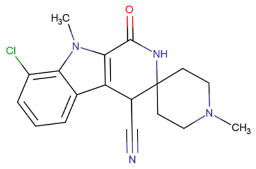

745321

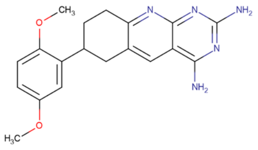

714363

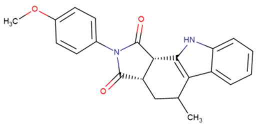

643416

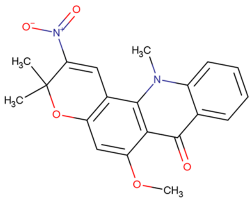

380856

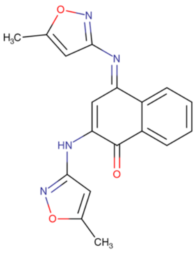

649750

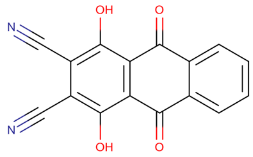

27034

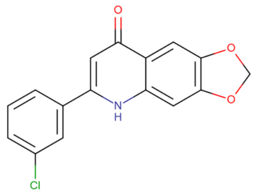

656160

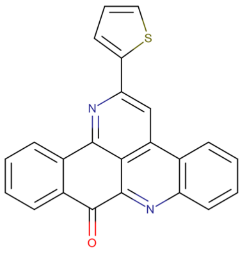

690040

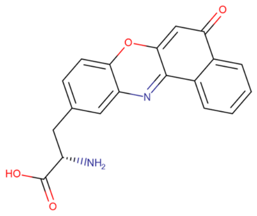

672425

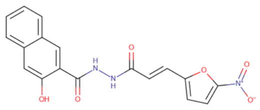

750908

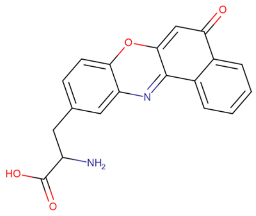

643735

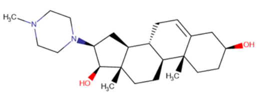

677959

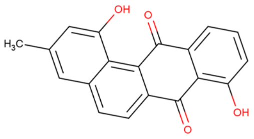

194618

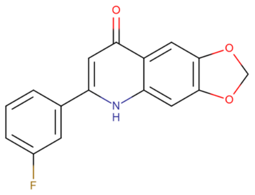

656159

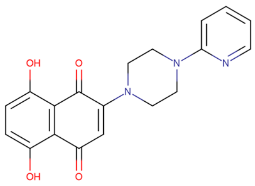

658142

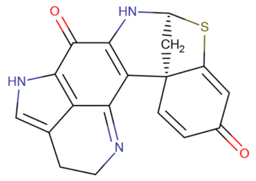

656204

*Listed are NSC-713198, NSC-667933, NSC-679749, NSC-707982, NSC-745321, NSC-714363, NSC-643416, NSC-380856, NSC-649750, NSC-27034, NSC-656160, NSC-690040, NSC-672425, NSC-750908, NSC-643735, NSC-677959, NSC-194618, NSC-656159, NSC-658142, and NSC-656204.

Figure S3. 2D structures of top 20 drug-like ligands for ovarian cancer.*

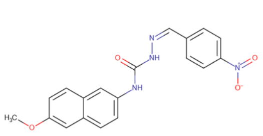

690273

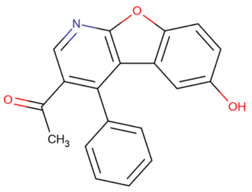

720028

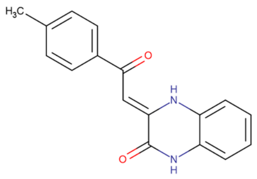

720447

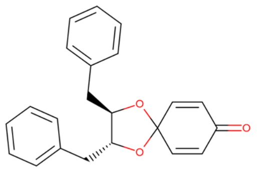

720702

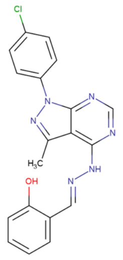

753018

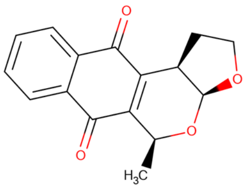

722392

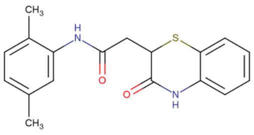

634398

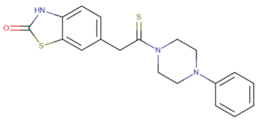

690678

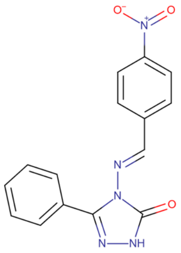

675223

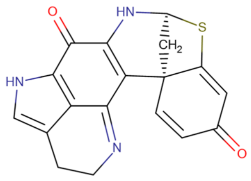

656204

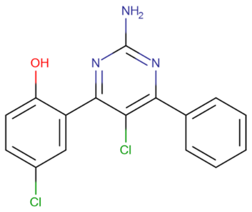

621457

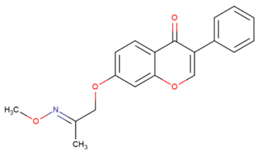

726393

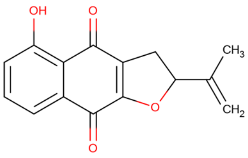

692229

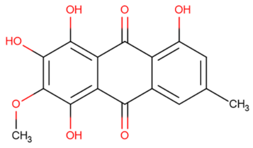

257450

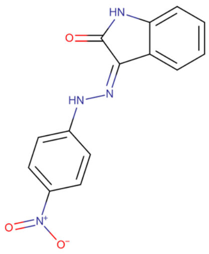

116536

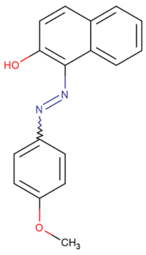

70811

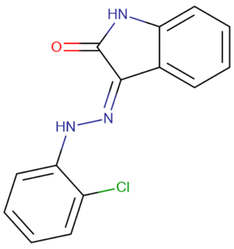

117192

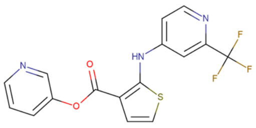

753598

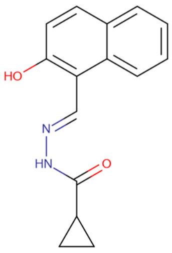

72881

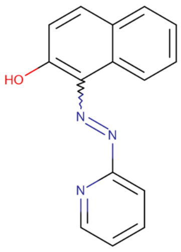

5332

*Listed are NSC-690273, NSC-720028, NSC-720447, NSC-720702, NSC-753018, NSC-722392, NSC-634398, NSC-690678, NSC-675223, NSC-656204, NSC-621457, NSC-726393, NSC-692229, NSC-257450, NSC-116536, NSC-70811, NSC-117192, NSC-753598, NSC-72881, and NSC-5332.

Figure S4. 2D structures of top 20 drug-like ligands for renal papillary cancer.*

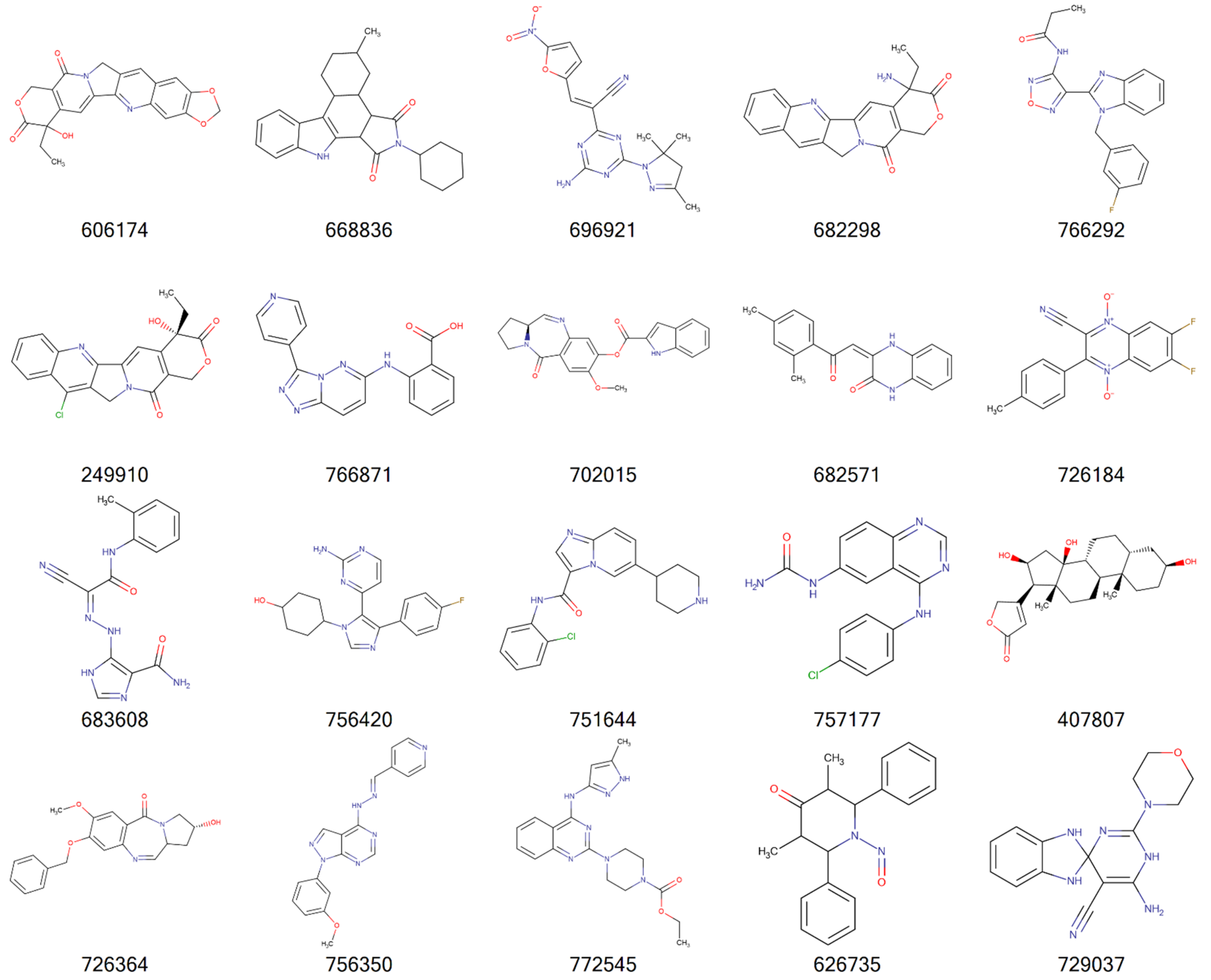

*Listed are NSC-606174, NSC-668836, NSC-696921, NSC-682298, NSC-766292, NSC-249910, NSC-766871, NSC-702015, NSC-682571, NSC-726184, NSC-683608, NSC-756420, NSC-751644, NSC-757177, NSC-407807, NSC-726364, NSC-756350, NSC-772545, NSC-626735, and NSC-729037.
